# Coronary artery disease incidence, risk factors, awareness, and medication utilization in a 10-year cohort study

**DOI:** 10.1186/s12872-024-03769-3

**Published:** 2024-02-12

**Authors:** Mohammadtaghi Sarebanhassanabadi, Seyed Reza Mirjalili, Pedro Marques-Vidal, Alexander Kraemer, Seyedeh Mahdieh Namayandeh

**Affiliations:** 1 Yazd Cardiovascular Research Center, Non-communicable Diseases Research Institute, Shahid Sadoughi University of Medical Sciences, Yazd, Iran; 2 Department of Internal Medicine, Rue du Bugnon 46, Rue du Bugnon 46, Lausanne, BH10-642, CH-1011, CH-1011 Switzerland; 3 Department of Health Sciences, Bielefeld University, Bielefeld, Germany; 4 Afshar Clinical Research Development Center, Yazd Cardiovascular Research Center, Non-communicable Diseases Research Institute, Shahid Sadoughi University of Medical Sciences, Yazd, Iran

**Keywords:** Coronary artery disease, Epidemiology, Yazd Healthy Heart cohort, Incidence, Prevalence, Risk factor, Awareness, Medication adherence

## Abstract

**Background:**

There is a substantial disparity in coronary artery disease (CAD) burden between Iran and other nations that place a strong emphasis on the assessment of CAD risk factors and individuals’ awareness and ability to control them.

**Methods:**

Two thousand participants of a community-based Iranian population aged 20–74 years were investigated with a mean follow-up of 9.9 years (range: 7.6 to 12.2). An analysis of Cox regression was conducted to determine the association between CAD development and classic risk factors such as age, sex, smoking, physical activity, education, obesity, dyslipidemia, hypertension, and diabetes mellitus. Furthermore, we computed the population attributable fraction for these risk factors.

**Results:**

After a follow-up period of nearly 10 years, 225 CAD events were reported, constituting 14.5% of the overall incidence. Nighty three percent of participants had more than one risk factor. Age was the most predictive risk factor, with a hazard ratio (HR) and confidence interval (CI) of 5.56 (3.87–7.97, *p* < 0.001) in men older than 45 and females older than 55 compared to lower ages. In comparison to females, males had an HR of 1.45 (CI: 1.11–1.90, p value = 0.006) for developing CAD. Nearly 80% of the patients had dyslipidemia, with a hazard ratio of 2.19 (CI: 1.40–3.44, *p* = 0.01). Among the participants, 28.9% had hypertension, and 52% had prehypertension, which had HRs of 4.1 (2.4–7.2, *p* < 0.001) and 2.4 (1.4–4.2, *p* < 0.001), respectively. Diabetes, with a prevalence of 17%, had an HR of 2.63 (CI: 2 -3.47, *p* < 0.001), but prediabetes was not significantly associated with CAD. Awareness of diabetes, dyslipidemia, and hypertension was 81%, 27.9%, and 48.1%, respectively. Regarding medication usage, the corresponding percentages were 51% for diabetes, 13.2% for dyslipidemia, and 41% for hypertension.

**Conclusions:**

Compared to previous studies in Iran and neighboring countries, the current study found a higher incidence of CAD, more prevalent risk factors, and a lower awareness and ability to control these risk factors. Thus, an effective preventive strategy is needed to reduce the CAD burden in Iran.

## Introduction

Globally, cardiovascular disease (CVD) is the leading cause of disability and premature death [1]. Coronary artery disease (CAD) emerges as the predominant form of CVD [2]. According to the global burden of disease (GBD) data, CAD was responsible for 16.2% of all-cause deaths and 7.19% of disability-adjusted life years (DALY) worldwide in 2019 [3]. Notably, over 75% of CVD deaths occur in low- and middle-income countries, indicating that CVD disproportionately affects low/middle-income countries [4].

GBD reports that Iran had 593,000 new CAD cases in 2019, accounting for 26.2% of all deaths and 10.3% of DALYs, higher than the global average. A 2014 study in Tehran, the capital city of Iran, found that men’s CAD incidence rate was 11.9 per 1000 person-years, while women’s was 6.5, similar to those in the United States in the 1970s [5]. In order to address this disparity, a systemic preventive plan should be established both at the community and individual levels.

The concept of prevention refers to a set of coordinated actions aimed at reducing or eliminating diseases and their disabilities [6]. The initial stage of this concept involves identifying and assessing the extent of the burden and its contributing variables. Hence, doing a thorough examination of the incidence of CAD and the prevalence of its associated risk factors in the population, as well as determining the individual contribution of each risk factor to the development of this illness, is an essential aspect in CAD prevention at community level and can significantly impact future actions [7]. Furthermore, a component of this concept pertains to the individual level, specifically if the person is cognizant of their risk factors and whether they take any proactive or therapeutic measures to address those risk factors [7].

Numerous studies have investigated the cardiovascular health situation in our region, revealing the prevalence of risk factors such as hypertension, obesity, diabetes, and dyslipidemia. In 2018, a review of cardiovascular disease surveys in the Eastern Mediterranean region revealed that Iran had a prevalence of 20.4% for hypertension, 12.2% for impaired glucose, 54.1% for dyslipidemia, 62.3% for overweight, and 26.1% for obesity [8], however, all of these statistics date back to before 2010. A national study conducted based on STEPwise approach to non-communicable disease risk factor surveillance (STEPS) in Iran in 2021, revealed the following prevalence rates of CAD risk factors: 51.3% of the population had low physical activity, 19.4% were smokers, 32% had hypertension, 63% had BMIs over 25, and 17.5% had diabetes [9]. However, these risk factors were not evaluated for their impact on CAD incidence. Although there have been several attempts to compile the burden of CAD in Iran in the past [2, 5, 10–12], there is no comprehensive analysis available that compares risk factors, their attributable risk, self-awareness of people about them, and medications use for risk factors in a single framework.

Based on the aforementioned findings, we conducted a prospective cohort study to ascertain the incidence of CAD, investigate the prevalence of risk factors linked to CAD, analyze the hazard ratios, calculate the population attributable fractions, assess individuals’ awareness of these risk factors, and evaluate the consumption of medication in relation to CAD risk factors.

## Methods

### Study setting

This cohort study was based on the Yazd Healthy Heart Project (YHHP), which evaluated cardiovascular diseases and metabolic disorders in a population-based setting. In this project, 100 clusters were selected based on the city’s geographic locations, and 20 families were identified in each cluster. One adult between the ages of 20 and 74 was selected randomly from each family for participation and evaluation (*n* = 2000, 1000 men, 1000 women). The Yazd Cardiovascular Research Center (YCRC) evaluated participants only twice, once during the study’s inception (2005–2006) and again after ten years (2015–2016).

### Ethical statement

The present study was approved by the Shahid Sadoughi University of Medical Sciences ethics committee (ethics code: IR.SSU.REC.1397.188) and conducted based on the Declaration of Helsinki on medical research [13]. Informed consent was obtained from the study participants during the initial and follow-up phases. The present research is based on strengthening the reporting of observational studies in epidemiology (STROBE) statement [14].

### Included participants

From the 2000 participants, 17 were eliminated from the study due to loss during the second phase; from the 1983 individuals participating in the baseline examination, 62 were excluded due to diagnosis of CAD at baseline, 78 due to death during the study, and 308 due to missing data [15]. The remaining 1552 participants (804 men, mean age 48.6 ± 14.7 years) were included in the present study. Participants were evaluated in both phases as outlined below.

### Clinical and biological data

Laboratory tests were performed after overnight fasting. We measured glucose and triglyceride (TG) after centrifugation using kits from Pars Azmoon Inc. (Tehran, Iran). Lipid profiles (total cholesterol, low-density lipoprotein [LDL], and high-density lipoprotein [HDL]) were analyzed with Bionic kits (Bionic Company, Tehran, Iran). The analyses were carried out using a biochemical autoanalyzer (BT 3000, Italy). Prediabetes was defined as fasting blood sugar (FBS) values between 100 mg/dl and 125 mg/dl. Diabetes was defined as FBS values ≥ 126 mg/dl or confirmed diabetes by a physician. Dyslipidemia was defined as TG ≥ 150 mg/dl or LDL ≥ 130 or HDL ≤ 40 in men or ≤ 50 in women or total cholesterol ≥ 200 mg/dl or medical treatment or confirmed dyslipidemia by a physician.

### Anthropometric features

The participants’ heights were measured using a stadiometer fixed to a wall with no dents or bumps. They stood barefoot, their heels, hips, shoulders, and heads touching the wall, and their heads were fixed horizontally. A 0.5 cm margin of error was used to measure the heights. Participants were weighed to the nearest 0.1 kg in the first phase using a digital scale (Seca, Germany) with minimal clothing and in the second phase using a body composition monitor and scale (Model BF511, Omron Co. Karada body scan, Osaka, Japan). The superior border of the iliac crest and widest part of the buttock were considered to measure waist and hip circumferences, respectively, to the nearest 0.1 cm using a nonstretchable tape. To be considered obese, a person had to have either a BMI > 30, a waist circumference > 94 cm in men or > 80 cm in women, or a waist-to-hip ratio > 0.9 in men or > 0.85 in women [16].

### Blood pressure measurements

A digital automatic blood pressure monitor (Omron, M6 comfort, Osaka, Japan) was used to measure blood pressure in participants’ right arms in a sitting position. Nursing staff measured blood pressure twice, with a 5-minute interval between measurements. Prehypertension was defined as systolic blood pressure ≥ 120 mmHg and < 140 mmHg or diastolic blood pressure ≥ 80 mmHg and < 90 mmHg. Hypertension was defined as systolic blood pressure ≥ 140 mmHg, diastolic blood pressure ≥ 90 mmHg or medication consumption for hypertension.

### Physical activity, education, awareness and medicine consumption

Questionnaires that were completed by trained interviewers were used to collect demographic information, education, physical activity, smoking habits, and angina pectoris. Educational attainment was categorized into primary, high school, and academic education categories. The International Physical Activity Questionnaire (IPAQ) [17] was used to assess physical activity. As part of this questionnaire, participants were asked about their number of days and the amount of time they spent walking, participating in moderate intensity activity, and participating in vigorous activity. According to these questions, an index called MET-hours per week is calculated that is equal to 1 kcal/kg/hr [18], and based on this index, participants were divided into low-, moderate-, and high-activity groups. Participants were divided into groups of smokers or nonsmokers based on their current smoking status. We asked participants about past medical history and drug history related to diabetes, dyslipidemia, and hypertension. As a result of these histories, we were able to assess whether these risk factors were known and how they were treated medically.

### Outcome definition

CAD events were defined as occurrences of fatal and nonfatal CAD, myocardial infarction (MI), percutaneous coronary intervention (PCI), coronary artery bypass grafting (CABG), and new angina pectoris. The diagnosis of new angina was based on positive findings from the Rose angina questionnaire in addition to positive electrocardiogram changes, elevated cardiac enzymes, and positive exercise tolerance test or coronary artery angiogram. Electrocardiogram findings were confirmed by two people, a general practitioner, and a trained nurse and confirmed by a cardiologist if controversies existed. According to medical records, the time of outcome for fatal and nonfatal CAD, MI, CABG, positive exercise tests, positive cardiac enzymes, and PCI was determined.

### Statistical analysis

Statistical analyses were performed with SPSS version 24.0 (IBM Corp., Armonk, NY, USA). Categorical variables were described as numbers (percentages) and were compared using chi-square tests. Continuous variables were described as mean and standard deviation and were compared using T tests. The incidence of CAD was calculated using the following equation:

$$Incidence/1000 person-year=\left(\frac{ New\, Cases}{Population \times Timeframe}\right)$$

Older age was defined as being older than 45 years in men and 55 years in women. Gender, smoking, physical activity, education, obesity, dyslipidemia, hypertension, and diabetes were analyzed as categorical variables. All categorical variables were analyzed in the total population and additionally based on sex-specific distribution. Cox regression was used to estimate the risk of CAD development, and the results were expressed as hazard ratios and 95% confidence intervals. Population attributable fraction (PAF) was calculated to determine the percentage of outcomes that could be prevented by removing a risk factor.

PAF= (P * (RR-1)) / (1 + P * (RR-1)).

P = The proportion of exposed subjects in the entire study population.

RR= (a / (a + b)) / (c / (c + d)).

For protective factors, that have a negative RR, the PAF is negative. Factors that did not show any significant effect on CAD were excluded from PAF analysis.

## Results

### Characteristics of participants

Table 1 presents a comprehensive summary of the diabetes, dyslipidemia, blood pressure, and obesity statuses of the participants who were included in the study in comparison to those who were omitted from the analysis. Other characteristics of the study participants according to the follow-up process were previously published. Included participants were older and significantly more male [15]. Men were heavier, smoked more, had higher educational levels, and had higher blood pressure, as well as lower HDL-cholesterol levels, total cholesterol, and LDL-cholesterol [15].

**Table 1:** Characteristics of the participants based on inclusion process at baseline

Variable	Included *N* = 1552	Excluded *N* = 448	*P* value
**Dyslipidemia**	1239(80.1)	323(73.2)	0.07
Total cholesterol	199.3 ± 45.1	198.3 ± 93.9	0.23
LDL^1^	109.2 ± 36.8	106.2 ± 35.1	0.4
TG^2^	179.8 ± 109.2	170.7 ± 103.1	0.29
HDL^3^	54.1 ± 13.8	53.2 ± 13.2	0.41
**Obesity**	1222(79.2)	345(78.4)	0.71
BMI^4^ (Kg/m^2^)	26.2 ± 4.3	26 ± 4.9	0.64
Waist circumference (cm)	93.8 ± 12.1	92.4 ± 12.6	0.64
Waist/hip ratio	0.9 ± 0.1	0.9 ± 0.1	0.73
**Blood pressure**			
**No**	297 (19.1)	101(22.5)	0.11
**Prehypertension**	807 (52.0)	213(47.5)	0.16
**Hypertension**	449 (28.9)	134(29.9)	0.87
SBP^5^	128.2 ± 15.5	127.9 ± 16.4	0.98
DBP^6^	82.7 ± 8.8	82 ± 9.2	0.15
**Diabetes**			
**No**	1065(68.6)	307(68.4)	0.63
**Prediabetes**	224 (14.4)	48(10.7)	0.15
**Diabetes**	264 (17)	93(20.8)	0.60
FBS^7^	103.1 ± 46	102 ± 44.2	0.92
**Smoking**	280 (18.1)	69 (15.6)	0.23

### Incidence of coronary artery disease

A total of 225 CAD events were reported after a follow-up period of 15,420.11 person-years. Of these, 135 events occurred in men and 90 in women. The 10-year cumulative incidence rate for males was 16.8 (14.4–19.2) per 1000 person-years, whereas for females, it was 12.0 (9.5–14.5). The incidence of new-onset CAD at the second visit was 14.5% (Table 2).

**Table 2:** Cumulative risk of CAD based on gender and total population

Sex	At risk (%)	Lost to follow-up (%)	Cases (%)	10-yr cumulative incidence rate (CI)
**Men**	804 (51.9)	152 (15.8)	135 (16.7)	16.8 (14.4–19.2)
**Women**	748 (48.1)	233 (23.7)	90 (12.0)	12.0 (9.5–14.5)
**Total sample**	1552	385 (19.8)	225 (14.5)	14.5 (12.4–16.6)

### Risk factors, awareness, and medication utilization

**Fig. 1:**
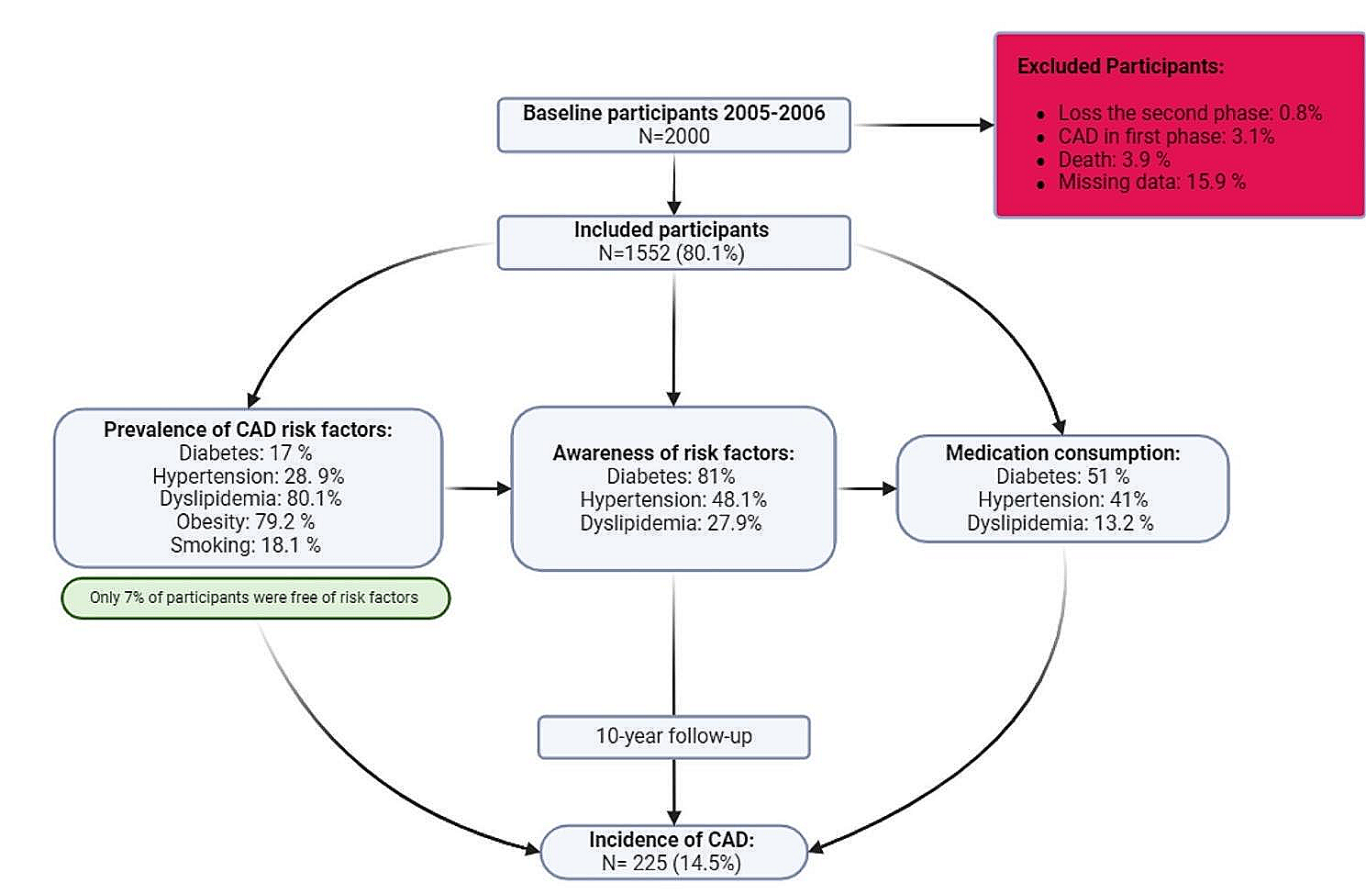
Flow diagram of participants inclusion and 10-year follow up

Figure 1 demonstrates follow-up process and prevalence of CAD risk factors. Only 7% of our population was free of CAD risk factors. 78% of the participants had two or more risk factors. The prevalence of diabetes, hypertension and dyslipidemia was 17%, 28.91%, and 80.1%, respectively, in the first phase. The rates of awareness for diabetes, dyslipidemia, and hypertension were found to be 81%, 27.9%, and 48.1%, respectively. In regard to the medication prescribed usage, the respective proportions were found to be 51% for diabetes, 13.2% for dyslipidemia, and 41% for hypertension.

### Contribution of risk factors in CAD development

Table 3 demonstrates the HR of different risk factors for the 10-year incidence of CAD. Older age had an HR of 5.56 (3.87–7.97, p value = 0.0001) compared to the lower age group. Male gender in comparison to female gender had an HR of 1.45 (CI: 1.11–1.90, p value = 0.006). Smoking was associated with a significant risk of CAD, with an HR of 1.42 (CI: 1.05–1.93, p value = 0.02). However, this association was not significant after gender stratification. Dyslipidemia, with an HR of 2.19 (CI: 1.40–3.44, p value = 0.01), was an important risk factor for CAD. Prehypertension had an HR of 2.38 (CI: 1.36–4.18, p value < 0.01), and hypertension had an HR of 4.13 (CI: 2.36–7.2, p value < 0.01). Diabetes had an HR of 2.63 (CI: 2-3.47, p value < 0.01), but prediabetes was not significantly related to CAD.

**Table 3:** Hazard ratio of CAD risk factors after 10-year follow-up

Variable	At risk (%)	Incidence/1000 person-year	Hazard ratio HR (95%CI)	*P* value	PAF* (%)
**Age group**					
Lower age	776	35	1	< 0.001	-
Older age	776	190	5.56 (3.9-8)		68.9
**Gender**					
Female Male	748(48.2)	11.7	1	0.01	-
	804(51.8)	17	1.4 (1.1–1.9)		16.9
**Smoking**					
Nonsmoker Smoker	1268(81.9)	13.3	1	0.02	-
	280(18.1)	20.1	1.4 (1.05–1.9)		8.4
**Physical activity**					
Low Moderate High	720(67.9)	19.8	1	0.0001	-
	290(27.4)	11.1	0.6 (0.4–0.8)		-14.4
	50(4.7)	5.9	0.27 (0.1–0.8)		-4.7
**Education**					
Low Moderate High	904(59.8)	19.7	1	0.0001	-
	460(30.4)	7.5	0.4 (0.3–0.6)		-26.5
	148(9.8)	9.5	0.45 (0.3–0.8)		-7.8
**Obesity**					
Non obese Obese	320(20.8)	8.3	1	0.01	-
	1222(79.2)	16.2	1.8 (1.2–2.7)		53.6
**Dyslipidemia**					
No Yes	307(19.9)	6.9	1	0.01	-
	1239(80.1)	16.5	2.2 (1.4–3.4)		64.6
**Hypertension**					
No Prehypertension Hypertension	297(19.1)	4.8	1	< 0.001	-
	807(52)	12.6	2.4 (1.4–4.2)		55.1
	449(28.9)	24.3	4.1 (2.4–7.2)		71.6
**Diabetes**					
Non diabetic Pre diabetes Diabetes	1064(68.6)	10.5	1	< 0.001	-
	224(14.4)	18	1.3 (0.9–1.8)		30.2
	264(17)	29	2.63 (2 -3.5)		32.5
**Total**		14.5	-	-	

*PAF; population attributable fraction percent

Women exhibited a significantly greater HR for hypertension and diabetes but had a lower hazard ratio for age (Table 4, p for interaction < 0.05). HTN, age, and dyslipidemia exhibited the three highest PAFs at 71.6%, 68.9%, and 64.6%, respectively. In men, age demonstrated the highest PAF at 84.6%, while in women, hypertension exhibited the highest PAF at 72.2%.

**Table 4:** Risk of CAD after 10-year follow-up stratified by gender

Variable	Men						Women					
	At risk (%)	Incidence/1000 person-year	Hazard ratio HR(95%CI)		*P*-value	PAF* (%)	At risk (%)	Incidence/1000 person-year	Hazard ratio HR(95%CI)	*P*-value	PAF* (%)	*P* for interaction
**Age groups**												
Lower age	327	13		1	< 0.001	-	449	22	1	< 0.001	-	< 0.001
Older age	477	122		7.35 (4.1–13)		84.6	299	68	4.2 (2.6–6.8)		53.8	
**Smoking**												
Non-smoker Smoker	529(66)	15.2		1	0.2	-	739(98.9)	12.1	1	0.8	-	-
	272(34)	20.3		1.2 (0.9–1.7)		-	8(1.1)	12.3	0.8 (0.1-6.0)		-	
**Physical activity**												
Low Moderate High	437(68.7)	21.6		1	0.001	-	283(66.8)	17.1	1	0.07	-	-
	161(25.3)	11.3		0.5 (0.3–0.8)		-14.6	129(30.4)	10.9	0.7 (0.4–1.3)		-	
	38(6)	7.9		0.31 (0.1-1.0)		-	12(2.8)	8.1	- (-)		-	
**Education**												
Low Moderate High	378(48.7)	24.9		1	0.001	-	526(71.5)	16	1	0.001	-	-
	279(36)	10.2		0.4 (0.3–0.6)		-33.5	181(24.5)	3.3	0.21 (0.1–0.5)		-25.4	
	118(15.3)	20.4		0.4 (0.2–0.7)		-14	30(4)	3.3	- (-)		-	
**Obesity**												
Non-Obese Obese	247(31)	9.1		1	0.001	-	73(9.8)	5.7	1	0.3	-	-
	550(69)	20.5		2.2 (1.4–3.5)		47.2	672(90.2)	12.7	1.8 (0.6–4.8)		-	
**Dyslipidemia**												
No Yes	178(22.2)	9.1		1	0.01	-	129(17.3)	3.9	1	0.01	-	0.23
	622(77.8)	19.2		1.9 (1.1–3.2)		46.3	617(82.7)	13.8	3.3 (1.3–8.2)		67.9	
**Hypertension**												
Normal Pre-hypertension Hypertension	106(13.4)	6.7		1	0.00	-	190(25.4)	3.7	1	0.00	-	-
	451(56)	14.2		1.9 (0.8–4.1)		-	356(47.6)	10.7	2.6 (1.2–5.9)		55.3	
	247(30.6)	26.3		3.2 (1.5-7.0)		67.8	202(27)	21.9	4.7 (2.1–10.4)		72.2	< 0.001
**Diabetes**												
Non-diabetic Pre-diabetic Diabetic	560(69.7)	12.6		1	0.001	-	498(66.6)	5.3	1	0.00	-	
	115(14.28)	23.9		1.8 (1.2–2.8)		12.7	115(15.4)	11.9	1.5 (0.8–2.9)		-	
	129(16.02)	27.4		1.93 (1.32–2.83)		18.7	135(18.05)	30.4	4.09 (2.7–6.19)		54.3	< 0.001
**Total**	804	16.8					748	12				

*PAF; population attributable fraction percent

## Discussion

The incidence of CAD in second phase was 14.5%. Aside from age and sex, which cannot be modified, dyslipidemia, hypertension, diabetes, and obesity were major risk factors in this study. Furthermore, physical activity and education had protective effects. Women were more likely to suffer from dyslipidemia, HTN, and diabetes, while men were more likely to be affected by CAD as a result of aging. Awareness of the CAD risk factors, especially dyslipidemia and HTN, and compliance with medications consumption were low. Previous research reported 12 and 9 occurrences of CAD per 1000 individuals in Iran [5, 19], our study uncovered a notably higher incidence rate over a 10-year period. In a comparative context, earlier studies conducted in different regions of Iran, such as Kerman and Bushehr, have highlighted the prevalence of CAD risk factors [10, 11]. For instance, research in Kerman indicated that 60% of the population had at least two CAD risk factors. Similarly, in Bushehr, 44.3% of males and 69% of females were found to have at least two risk factors. These findings are somewhat lower than those observed in current study, suggesting regional variations in risk factor prevalence within Iran. Additionally, when comparing our findings with international studies, such as one conducted in China, the differences become more pronounced. The Chinese study revealed that 31.1% of participants did not have any identified CAD risk factors [10], a proportion 4.4 times greater than what we observed in our Iranian cohort. This stark difference may be attributable to a combination of genetic, cultural, lifestyle, and dietary differences between the populations.

### Dyslipidemia

Dyslipidemia was the most prevalent CAD risk factor in our study. According to world health organization (WHO) reports, it is associated with half of all ischemic heart diseases [8]. In this regard, we found that dyslipidemia can increase the risk of CAD by 64.6%. When compared to other Iranian provinces, our study area exhibited a notably higher prevalence of dyslipidemia (80%), surpassing rates in Lorestan (64%), Kerman (26%), and Isfahan (70%); only Mashhad showed a slightly higher rate at 83.4% [10, 11, 20]. This trend extends beyond Iran, with dyslipidemia prevalence reported at 69% in the UAE, 79.6% in Turkey, and 33% in Saudi Arabia [8, 20]. Intriguingly, our study also revealed the lowest rate of medication use for dyslipidemia in the Eastern Mediterranean Region (EMR), with just 13.2% compared to Lebanon’s (second from last country) significantly higher 41.2% [8].

According to that, the retention of LDL and other cholesterol-rich apolipoprotein (Apo) B containing lipoproteins in the arterial wall is the initial cause of atherogenesis [21] and lowering LDL values reduces the risk of future cardiovascular events, with no minimum threshold [7], it becomes imperative to enhance the screening and treatment of dyslipidemia in our population. This strategy is crucial, considering the high prevalence and low medication use observed, to effectively mitigate the CAD risk.

### Smoking

A smoking prevalence of 18.4% was found in the current study, which was lower than what was reported in 2015 in the EMR [8]. Despite its widespread prevalence and its confirmed role as a risk factor, smoking was not significantly associated with CAD in our population. Although the association was significant in the total population, it lost its significance when analyzed separately by gender. This may be due to bias caused by self-report and qualitative data collection that resulted from the fear of social and cultural stigmatism of smoking (reporting bias), particularly in women.

Smoking stands apart from other risk factors due to its ability to additionally affect people through passive smoking, which is an independent and distinct cause of CVD [22]. It is estimated that 41,000 deaths per year occur in USA is due to secondhand smoke [23]. Smoking has been found to have a dose-response relationship with the development of CAD. For instance, individuals who smoke fewer than 20 cigarettes per day have a relative risk (RR) of 2.15, while those with > 20 cigarettes per day have a RR of 3.28. Additionally, passive smokers have a RR of 1.31 for developing CAD [24].

According to the notable decline in CAD cases in the USA from 1970 to 2015, which has been partly attributed to reduced smoking rates [25], it is essential to address smoking as a key strategy in reducing CAD incidence in our society. This includes addressing cultural attitudes and behaviors towards smoking through the use of social media.

### Obesity

In 2015, the United States experienced a concerning 1% increase in mortality due to CAD [26], marking an end to a four-decade trend of declining CAD deaths. This recent rise has been primarily linked to the increasing prevalence of obesity [27]. The Framingham study highlighted this by demonstrating that obesity elevates the likelihood of CAD by 64% for females and 46% for males [10]. Our study supports these findings, indicating a 53.6% increased risk of CAD in the total population.

In Iran, the national obesity prevalence was reported at 63% [9], which is notably lower than the rates observed in our study. This disparity could be attributed to our comprehensive approach in assessing obesity, which included waist circumference, BMI, and waist-hip ratio measurements. This method is recognized as the most effective for evaluating obesity-related risks [27].

According to the definitions of obesity provided by international diabetes federation (IDF) [28] and WHO [16], 69% of men and 90% of women were classified as obese. However, the association between obesity and CAD was found to be significant only in men after separating the data by gender. This suggests that the definitions of obesity may need to be revised specifically for Iranian women, as there is a high rate of false positive predictions of CAD.

### Diabetes

The diabetes prevalence rate was 17%, leading to a notable rise in the occurrence of CAD. However, considering that an estimated four out of ten adults with diabetes in the EMR are currently undiagnosed [8], it is plausible to suggest that the diabetes prevalence in our population may be around 28%. The Yazd Healthy Study (YaHS) reported a prevalence of 14.1% in 2020 [29]; this difference in prevalence with the current study is probably because only 40% of their study participants had blood samples. There was a higher level of awareness (80%) and drug consumption (50%) for diabetes when compared with other risk factors, but in EMR, Kuwait is ahead with 85.4% medication use for diabetes [8].

Diabetes was significantly associated with CAD in both sexes, while prediabetes was only significant in men. In contrast, previous studies have demonstrated that borderline glucose tolerance groups have a 1.65 higher CAD risk [30]. Our study’s potential limitation in not including postprandial glucose levels, which are more strongly associated with CAD risk in prediabetic subjects [33], could explain this discrepancy [31]. Moreover, in a meta-analysis of prospective studies, prediabetes was associated with CAD in both sexes at the same OR [32]. Additionally, prior studies have demonstrated that hyperglycemia, even without a diabetes diagnosis, increases CAD risk and complications [33, 34].

Given these findings, it’s clear that diabetes is a significant public health issue, extending beyond the scope of medication prescription. To effectively manage diabetes and its impact on CAD, a comprehensive approach is needed. This approach should consider not only medical treatment but also factors like environmental influences, mental health (including depression and stress), self-efficacy, and social support [27]. Such evaluations are critical for improving glycemic control and ensuring adherence to treatment, thereby potentially mitigating the CAD risk associated with diabetes.

### Hypertension

In the present investigation, hypertension emerged as the primary modifiable risk factor for CAD, contributing to 71.6% of the incidence of CAD. Hypertension is also the leading modifiable risk factor responsible for the majority of CVD-related deaths in the United States [35]. Globally, the EMR is the second most prevalent region for HTN [8]. Among these countries, we had the highest prevalence of HTN (29.5%), which was higher than that in Somalia (26.4%). Alarmingly, our population also demonstrated the lowest rate of medication use for HTN at just 40.9%, lower than Pakistan’s 52.9% [8]. This underuse of medication, coupled with the common misperception among patients about correct medication usage [36], suggests that the actual burden of HTN may be greater than reported.

Our analysis further revealed a significant association between HTN and CAD in both sexes. However, prehypertension was notably linked with CAD exclusively in women, with females exhibiting a higher hazard ratio for CAD due to HTN. This gender-specific impact aligns with findings from a study in Tehran [37], indicating that women are potentially more susceptible to developing CAD from elevated blood pressure. Such gender differences in HTN’s impact on CAD risk underscore the potential utility of gender-specific blood pressure cutoff points for individual CAD risk assessment [38]. These insights into HTN’s role in CAD, particularly regarding gender differences and medication usage, highlight the need for increasing community awareness about HTN.

### Physical activity and education

In the United States, a significant health concern is that approximately half of the adults fail to meet the minimum requirements for physical activity [39]. This issue is even more pronounced in the EMR, which reports the highest global prevalence of insufficient physical activity. Within the EMR, Saudi Arabia stands out for having the region’s lowest levels of physical activity [8]. According to the Saudi Health Information Survey, 46.0% of men and a striking 75.1% of women engage in low to zero physical activity [8]. Interestingly, in our study, men exhibited even lower levels of physical activity than those reported in Saudi Arabia, emphasizing a critical area for public health intervention.

We discovered that engaging in moderate and vigorous physical activity significantly reduces the risk of CAD. While the focus of most guidelines is on aerobic exercise, resistance training should not be overlooked, as it can also lower blood pressure and improve glycemic control. The relationship between physical activity and reduced CAD risk appears to be linear and dose-responsive, with no evident threshold for benefits [40, 41]. This evidence strongly supports the need for initiatives aimed at enhancing and maintaining physical activity levels [40].

Furthermore, our study, aligning with previous research [8], indicates a significant association between higher education levels and reduced CAD incidence. However, it raises an important question: Does higher education necessarily translate into better knowledge and implementation of healthy lifestyles, proper nutrition, and consistent physical activity? This potential gap in translating education into practical health benefits warrants further investigation, as understanding and addressing it could play a crucial role in CAD prevention strategies.

Our study’s findings open up various opportunities for future research in CAD epidemiology and prevention. To begin, there is a clear need for longitudinal research with bigger, more diversified populations, including both rural and urban locations. Another important topic of research could be to look into the influence of educational interventions on lifestyle changes and CAD risk factors, particularly in populations with low health literacy. Furthermore, the impact of emerging risk factors, including environmental contaminants and psychosocial stresses, in CAD development requires additional exploration.

### Strengths and limitations

Our study offers the following strengths. First, this study was conducted prospectively in a community base population and results were determined in a definite manner, there is little chance of reverse causality or recall bias. As a result of combining the Rose angina questionnaire with ECGs, cardiac enzymes, exercise tolerance tests, and coronary angiograms, we were able to define CAD more precisely than previous studies. Unlike previous studies that recruited mostly middle-aged and older adults, we included both old and young participants. The duration of the follow-up period may have captured the overall risk of CAD during a person’s lifetime. However, it is important to note that this duration could have also influenced the outcomes due to our inability to regulate and assess volunteer health check-ups and lifestyle modifications.

The study was restricted to a single urban center, reducing generalizability and introducing urban bias. As a result of just one baseline investigation of risk factors, we may be prone to intraindividual variances in the results. There was a high likelihood of misclassification, reporting bias, and non-response bias of categorical variables, such as physical activity, smoking, and education, due to their self-reported nature. However, previous studies with better classifications, such as determining physical activity with an accelerometer, found similar results [41]. Despite experiencing a population decline of around 20% during the follow-up period, we did not see any significant differences between the individuals who were included and those who were excluded. This finding reduces the likelihood of attrition bias.

## Conclusion

The results of the current study were consistent with previous studies about the impact of different traditional risk factors. However, the higher incidence rate of CAD, more prevalent risk factors, lower self-awareness of these risk factors, and lower medication use compared to previous studies in Iran and neighboring countries may alarm Iranian health care policymakers regarding the need for an effective preventive strategy to reduce the burden of CAD.

## Data Availability

The datasets used and/or analyzed during the current study are available from the corresponding author upon reasonable request.

## Footnotes

1 Low density lipoprotein.

2 Triglyceride.

3 High-density lipoprotein.

4 Body mass index.

5 Systolic blood pressure.

6 Diastolic blood pressure.

7 Fasting blood sugar.

## Abbreviations

CAD: Coronary artery disease
CI: Confidence interval
YHHP: YAZD healthy heart project
TG: Triglyceride
LDL: Low-density lipoprotein
HDL: High-density lipoprotein
FBS: Fasting blood sugar
SUA: Serum uric acid
IPAQ: International Physical Activity Questionnaire
SD: Standard deviation
BMI: Body mass index
ECG: Electrocardiogram
PAF: Population Attributable fraction
Apo: Apolipoprotein
WHO: World health organization
IDF: International diabetes federation
EMR: Eastern Mediterranean region
